# A multi-institute, follow-up, observational study measuring nursing students’ adherence to infection prevention and control protocols in Saudi Arabia

**DOI:** 10.3389/fmed.2023.1282723

**Published:** 2024-01-11

**Authors:** Marwa Albarmawi, Lourance Al Hadid, Rafi Alnjadat, Ahmad Aljabery

**Affiliations:** ^1^Department of Nursing, Al-Zaytoonah University of Jordan, Amman, Jordan; ^2^Department of Nursing, Al-Balqa Applied University, Al-Salt, Jordan

**Keywords:** nursing student, infection prevention and control, hand hygiene, adherence, infection

## Abstract

**Background:**

Nursing students learn principles of infection prevention and control (IPC) and hand hygiene (HH) in clinical courses, and their learning is reflected in their practice.

**Objectives:**

The knowledge, attitude, and practice of principles of IPC and HH of the students were measured prior to and after attending an educational workshop. The adherence of the students to the IPC and HH protocols at the hospital was also observed.

**Methods:**

This study included a pretest-posttest time series follow-up and an observational part. During the first part of the study, students attended a workshop, which was preceded by a pretest. It was then followed by a posttest directly after finishing the workshop and in 12 weeks. Participants were submitted to an observational part by a trained observer to document certain skills taught earlier during the workshop.

**Settings:**

Students from three nursing schools in Saudi Arabia participated in the study.

**Participants:**

A total number of 130 completed the study protocol, and 100 completed the observation part.

**Results:**

Students were found to experience an improvement in their knowledge, beliefs, and commitment scales after the workshop. The attitude scale remained relatively unchanged over different tests. Most students performed the skills properly and adequately, but some failed to perform certain skills, like hand rub, and the proper use of disinfectants.

## Introduction

Hospital and healthcare system-acquired infections are preventable conditions that increase the cost of healthcare and risk patient safety (1). These infections might cause diseases in hospitals leading to high morbidity and mortality rates (2). Nursing students learn principles and protocols of infection prevention and control and hand hygiene in clinical courses to ensure the safety of the students and the patient (3). Educators teach these protocols in the preparatory laboratories before commencing clinical training. However, it is very challenging for nurse educators to follow students’ adherence to IPC and HH as they practice. Given that students develop clinical knowledge and skills while studying and transfer them to work after graduation, it is necessary to emphasize IPC and HH during this period to decrease the prevalence of nosocomial infections and improve patient safety (4, 5). Therefore, nursing students must receive focused training on IPC, which should also be followed throughout their study. Many studies report cross-sectional findings, and a very limited number followed the adoption of IPC over time. There is a need to have studies that provide empirical evidence on how students perform regarding IPC adherence.

Globally, the World Health Organization reported that hospital-acquired infections are among the main challenges increasing mortality and morbidity rates (6). Approximately 1.5 million people worldwide suffer from complications related to nosocomial or hospital-acquired infections (6). The reported rates of preventable hospital-acquired infections in developing countries are approximately 10% of all types of infections and could be as high as 37% in intensive care units (7). In Saudi Arabia, the reported rate of healthcare-associated infections was approximately 15% [851 infections among 5,523 hospitalized patients with an average hospital stay of 9 days (8)]. Another Saudi Arabian study from February 2007 to January 2008 reported the hospital and healthcare system acquired infection was 16 in every 1,000 patient days (9). A recent Turkish study showed that the incidence of hospital-acquired infections was between 7 and 10%, which also indicated that the cost of treatment for these infections was significantly high (10). Despite differences in the numbers from different countries, all reported rates agree on the importance of continuing efforts to minimize the serious impact these infections have on the well-being of health professionals and patients alike. There have been many outbreaks, including the coronavirus (e.g., COVID-19) and swine flu in recent years, and these continue to escalate the risk of fatal cross-infections among health professionals and patients. Thus, the prevention of infectious diseases has become a major concern to health professionals, researchers, and educators, which also include trainees and students from different health specializations (3). Compliance with IPC protocols is a necessity mandated by the devastating results reported on lack of compliance (11).

Compliance with IPC and HH protocols is usually an outcome of focused and adequate student education and training (12). Once students become practitioners, they are expected to apply what they learned emphasizing the best available practices in their daily activities, including IPC and HH measures. While student training on these measures occurs during their undergraduate training, the real impact of this training appears after graduation as they commence their real job as nurses (13). Consequently, IPC and HH protocols should be emphasized during undergraduate education where students are trained to practice ideally.

The recommendations have been made on IPC and HH measures for both nurses and nursing students, which further emphasize the need for follow-up to ensure continuity of the implementation of the IPC and HH protocols (14). However, there is a paucity of follow-up studies on the structuring, implementation, or evaluation of IPC programs among students, especially in the Middle East. With the presence of evidence on low levels of compliance to IPC and HH among healthcare workers and the high prevalence of nosocomial infection in different areas, we conducted the present study.

## Aims

This study was designed to achieve the following aims:

1. Measure nursing students’ knowledge, attitude, and practice of protocols of infection prevention and control and hand hygiene protocols before attending the workshop.
2. Measure the effectiveness of the educational workshop about protocols of infection prevention and control and hand hygiene on students’ compliance to these protocols over time, including retention and implementation.
3. Observe using random checks students’ adherence to the IPC and HH protocols at the hospital, including hand rub, use of gloves, and disposal of sharp and non-sharp wastes.

## Design

This study was constructed to be conducted in two parts: a pretest-post-test, a time series follow-up interventional part; and an observational part. During the first part of the study, students attended a workshop, which was preceded by a pretest. It was then followed by a post-test directly after finishing the workshop. After 12 weeks, the participants completed a second post-test, which was composed of the same questions in the previous tests. Participants were submitted to an observational part by two trained observers to document certain skills taught earlier during the workshop.

## Sample and settings

The sample size was decided using G*Power v.3.1.9.4, and 85 students with medium effect size 0.25, alpha 0.05, and power 0.95. All potential students were recruited considering the possible low response rate of approximately 53–67% (15). 130 students completed the study protocol, and 100 completed the observation part.

This study was carried out in a four-year undergraduate bachelor’s nursing program in three academic institutes in Western Saudi Arabia. The average number of students in each program is 150 with a range of 35–55 students for each level.

## The training workshop

The training workshop covered the basics of IPC guidelines and practices, including common resistant pathogens, practical applications within the hospital environment to avoid contamination and cross-infections, types, and methods of isolation, treating accidental sharp inoculation, waste management, and the types and uses of disinfectants. The workshop material was provided to students before the workshop in the form of an electronic version and was sent to their mobile phones. The workshop took 8 h and was divided into two lab training days.

## The procedure

Ethical approval was obtained from the Ethics Committees of the participating institutes complying with the local ethical rules and regulations within the participating universities in the study based on the ethics approval granted by Alghad International Colleges for Applied Medical Sciences (AICAMS-346/RN/2018). Eligible students were recruited from two academic programs by contacting the nurse educators and asking for permission to include the IPC and HH workshop during the laboratory weeks before commencing hospital-based clinical rounds, including the pretest and the post-tests. Students were then approached during the laboratory preparatory sessions. Out of seven, students from two programs participated in the study. Students were asked whether they might be interested in participating in the study, which was explained to them. It was emphasized that participation was voluntary and no names or any identifying characteristics would appear at any time during the study or in the dissemination process.

The total number of students on the visited courses was 130. All students agreed to attend the workshop and complete the pretest and post-tests. For the observational part, 100 students approved of being observed blindly and signed consent for this purpose; 30 students did not want to be observed during their presence in the clinical settings. The students, who accepted being observed, were ensured that their grades would not be influenced by the results from the observation; this observation was accomplished for research purposes. It was also emphasized that the results would be used to improve practice and not to punish any student. Students were informed that they would be observed by two trained nurses from each program, who were non-identifiable to them. It was also explained that they would not be aware of when and where they would be observed. Students agreed to this procedure and signed a consent.

All inquiries were answered regarding the study before attending the workshop. Further, students were informed that their responses on the pretest and the post-tests would not influence their course grades. Data were collated at the beginning of the workshop (pretest), after finishing the workshop with a 60-min gap (post-test I), and after 12 weeks from the workshop (post-test II). It was assumed by the researchers that 12 weeks, which represented the midpoint of the academic semester, is adequate for students to practice the skills in their clinical courses.

The IPC and HH training workshops were conducted on students enrolling in the following courses: adult, acute adult (critical care and emergency nursing), and intensive care courses. All workshops were conducted for students in the vicinity of the institutes by one of the researchers. On the day of the workshop, all students attended the pretest and then took a break for 30 min before joining the workshop, which took approximately 4 h. They took a coffee break for 30 min and then completed post-test I, which contained the same items as the pretest.

For the second part, all observers were trained using the study manual, which was developed by the researchers to reflect the items being observed. The training took place in the simulation lab of the college where the trainee observed one of the researchers performing the checklist skills while completing the study checklist. This checklist was evaluated by another researcher and an external observer (who was a clinical course nurse coordinator from the college) for the accuracy of the trainee check of the list. The acceptable level of accuracy of the trainees exceeded 90%. The checklist was developed for the study by the researchers based on the literature and was revised and approved by three clinical course coordinators for adherence with the clinical courses’ components, adequacy, and suitability.

## Data analysis

Data were analyzed using the Statistical Package of Social Science (SPSS, Chicago, IL, USA, version 21). Normality tests were used to establish the normal distribution of the data and to allow for the adoption of the parametric tests in the analysis. Descriptive statistics including means, standard deviation (SD), and frequency (n) were used to describe the sample characteristics and achievements on the study scale and answer research aims one and two. This type of analysis identified students’ level of knowledge, attitudes, beliefs, and commitment to principles of infection control and prevention, which were taught during the workshop. The study also identified students’ compliance using an observational checklist on different occasions. Students consented to the blind observation of principles of infection and prevention taught earlier during the workshop, which was separated from the pretest, and post-test. Pre-test, post-test I, and post-test II scores were analyzed using descriptive statistics. A one-sample *t*-test was performed to measure differences based on the academic year. The qualitative part was analyzed using manual frequency calculation by the observers and was checked by another observer for the accuracy of frequencies and percentages. A convenience sampling technique was used with sample size estimated by using G* power analysis (version 3.1.9.7). The required sample size using the *t*-test was 112 (using alpha 0.05, a medium effect size of 0.25, and a power of 0.80).

## Results

The sample consisted of 130 nursing students in the second, third, fourth, and fifth semesters of their study and aged between 18 and 32 years (Table 1). Students in this study finished the first year (two academic semesters). They had finished the following courses: the English language, basic sciences (like biology, anatomy, and physiology), introduction to nursing, and the fundamentals of nursing. During the first semester of the second year, they attended the adult nursing, physical assessment, pharmacology, and pathophysiology, which prepared them to start their clinical courses at the hospital that included performing a physical assessment, preparing, and administering medications, and participating in changing the dressing and other bedside nursing responsibilities. Although these duties were carried out under strict observation at the beginning, this observation becomes less as students advance in their studies. Students’ level of study was distributed in four different semesters (Table 1). The number of male students was higher than that of females. More than 60% (*n* = 80) of the students had a higher secondary school, scientific stream.

**Table 1:** Characteristics of the participants (*n* = 130).

Factor			
Age	Mean: 22.76	SD: 3.76	Range: 18–32 years
Sex	Male	72 (55.4%)	
	Female	58 (44.6%)	
Academic semester/year	Second/2nd year	30 (23.1%)	
	First/3rd year	36 (27.7%)	
	Second/3rd year	35 (26.9%)	
	First/4th year	29 (22.3%)	
High school stream	Scientific	80 (61.5%)	
	Health	50 (38.5%)	

Students completed a pretest, which examined their knowledge of IPC and HH protocols. It is necessary to mention that the material of the IPC and HH pretest has already been given to the students in the previous semester, and this exam tested the knowledge retained by the students. In addition, they attended a 6-h workshop at the beginning of the current semester, which was followed by post-test I. Additionally, students attended post-test II after 12 weeks after post-test I. Test items were similar in the three sets; however, questions and responses were shuffled.

Data were submitted to tests of normality, and they did not violate the standards of normal distribution. Therefore, parametric tests were adopted in the analysis of the study results.

It was found that the students’ mean scores on the knowledge scale improved significantly in both post-test I and II (Table 2). This improvement also includes increases in the mean scores on the beliefs and commitment scales. The mean score on the attitude scale remained relatively unchanged over the tests (i.e., pretest, post-test I, and post-test II). There were no statistically significant differences found among the students across the academic years in the pretest, post-test I, and post-test II (Table 3).

**Table 2:** Comparing student performance on the study scales in the pre-test, post-test I, and II.

	Pre-test			Post-test I			Pre/post-test I difference	Post-test II			Pre/post-test II difference
	Mean	SD	Range	Mean	SD	Range	Sig	Mean	SD	Range	Sig
Knowledge*	12.71	3.99	3–24	24.79	4.26	11–44	0.02	26.95	5.58	17–36	0.01
Attitude	25.89	5.96	7–49	25.79	5.09	7–44	0.15	26.85	6.11	7–49	0.04
Belief	35.22	9.27	7–49	38.85	8.60	14–49	0.03	40.59	8.06	22–49	0.01
Commitment	65.12	14.74	10–90	71.23	13.40	40–90	0.02	77.19	71.72	30–80	0.01

*The maximum score is 45.

**Table 3:** Students’ achievement in the pretest, post-test I, and post-test II based on the academic year.

	Academic year	Pretest		Post-test I		Post-test II	
		Mean	Sig*	Mean	Sig*	Mean	Sig*
Knowledge	2nd year	12.82	0.11	25.39	0.06	27.12	0.16
	First/3rd	14.56		25.89		27.89	
	Second/3rd	12.19		24.90		27.76	
	4th year	12.73		24.26		27.52	
Attitude	2nd year	25.27	0.09	26.96	0.12	26.91	0.32
	First/3rd r	26.11		28.04		26.78	
	Second/3rd	26.21		27.46		26.49	
	4th year	25.55		27.36		25.93	
Belief	2nd year	35.49	0.06	36.21	0.08	40.18	0.07
	First/3rd r	36.44		36.33		39.44	
	Second/3rd	35.85		37.09		40.00	
	4th year	34.69		37.84		41.38	
Commitment	2nd year	660.61	0.14	679.39	0.06	743.64	0.07
	First/3rd r	666.33		668.44		724.44	
	Second/3rd	667.88		663.88		744.46	
	4th year	659.92		659.96		729.94	

^*^Sig: level of significance <0.05.

Students (*n* = 100) were then observed for a set of predefined skills, which reflected the learned protocols in the workshop and emphasized during their clinical training. Using a single-blind method, two trained observers documented the observed skills while students were busy working with patients. The use of documentation from two observers was adopted to increase the inter-rater reliability and to ensure a higher level of accuracy (16). The two observers met upon finishing the observation session, discussed their documents, and agreed on each document. One hundred students from different academic levels were observed on four different occasions for their adherence to using proper techniques and skills of infection prevention and hand hygiene. These skills included rubbing hands with alcohol before and after contacting patients, using gloves correctly (according to the adopted guidelines), disposing of needles, using disinfectants, and disposing of both sharp and non-sharp wastes.

Students from the second semester (*n* = 26), the first semester of the third year (*n* = 29), the third year (*n* = 28), and the first semester of the fourth academic year (*n* = 17). There were no statistically significant differences among all four academic levels based on their level of adherence and compliance with the infection prevention and control and hand hygiene protocols. Students were observed during the third, fifth, eighth, and tenth weeks of their clinical rounds, which usually lasted for 14 weeks. The final 4 weeks of the semester were avoided as they included student clinical examinations and evaluations.

Student observation was performed by trained nurses using a checklist that contained the items under observation rated on three ratings: performed, incompletely performed, and not performed at all. Most students performed the skills properly and adequately. However, some students missed one or more minor issues as they were providing their care to patients. It was found that nearly one-third of the students (27%, *n* = 27) failed to perform hand rub with alcohol properly before contacting patients (Table 4). The number of students who did not comply with hand rub after contacting the patients on all four occasions, ranged between 31% (*n* = 31) and 55% (*n* = 55).

**Table 4:** Findings from the observation (*n* = 100).

Item	Done	Inadequately done	Not done at all
Rubs with alcohol/wash hands before contacting the patient 1	71 (71%)	27 (27%)	2 (2%)
Rubs with alcohol/wash hands before contacting the patient 2	81 (81%)	18 (18%)	1 (1%)
Rubs with alcohol/wash hands before contacting the patient 3	74 (74%)	21 (21%)	5 (5%)
Rubs with alcohol/wash hands before contacting the patient 4	70 (70%)	23 (23%)	7 (7%)
Rubs with alcohol/wash hands after contacting the patient 1	69 (69%)	26 (26%)	5 (5%)
Rubs with alcohol/wash hands after contacting the patient 2	56 (56%)	31 (31%)	13 (13%)
Rubs with alcohol/wash hands after contacting the patient 3	61 (61%)	27 (27%)	12 (12%)
Rubs with alcohol/wash hands after contacting the patient 4	45 (45%)	39 (39%)	16 (16%)
Using gloves correctly 1	79 (79%)	0 (0%)	21 (21%)
Using gloves correctly 2	89 (89%)	0 (0%)	11 (11%)
Using gloves correctly 3	83 (83%)	0 (0%)	17 (17%)
Using gloves correctly 4	55 (55%)	0 (0%)	45 (45%)
Disposing of needles correctly 1	88 (88%)	0 (0%)	12 (12%)
Disposing of needles correctly 2	61 (61%)	0 (0%)	39 (39%)
Disposing of needles correctly 3	66 (66%)	0 (0%)	34 (34%)
Disposing of needles correctly 4	54 (54%)	0 (0%)	46 (%)
Using disinfectant appropriately 1	68 (68%)	17 (17%)	15 (15%)
Using disinfectant appropriately 2	45 (45%)	25 (25%)	30 (30%)
Disposes of medical waste correctly (sharp) 1	63 (63%)	0 (0%)	37 (37%)
Disposes of medical waste correctly (sharp) 2	63 (63%)	0 (0%)	37 (37%)
Disposes of medical waste correctly (non-sharp) 1	67 (67%)	0 (0%)	33 (33%)
Disposes of medical waste correctly (non-sharp) 2	77 (77%)	0 (0%)	23 (23%)

Another interesting finding in this part deals with donning and using gloves correctly. The number of students complying with the proper donning and use of gloves kept declining over time; the earliest documented accounts of this skill had significantly higher levels of compliance as compared to the four sets of observation. Similar findings can be seen in the disposal of used needles. Alarmingly, more than one-third of the students failed to dispose of used needles correctly on two different occasions, 37% (*n* = 37) and 33% (*n* = 33), respectively. Students also had issues with using disinfectants, which was observed on two different occasions. Students, who did not use disinfectants or did not use them properly were 32% (*n* = 32) for the first time, but the number increased to become 55% (*n* = 55) in the second observation time. In addition, approximately one in every three students (33%, *n* = 33) for the first time, and one in every four students (23%, *n* = 23) for the second observation time failed to comply with the guidelines when disposing of non-sharp wastes.

## Briefing students and educators

As part of the benefits of this study, all students and educators were gathered and briefed about the results. Data were analyzed and the results were arranged in a single report, which was provided to the departments of the institutes involved in this study. All students were briefed about the findings, and the main issues of concern were discussed as corrective measures for students, who did not comply with all infection prevention and control protocols or complied variably. The discussions took place at the end of the semester and no identifying features were discussed; results were presented as a single set of data that represents all institutes.

## Discussion

Results in this study demonstrated that nursing students’ level of knowledge of IPC and HH was low in the pretest. Similar results can be found in other studies (17–19). In contrast, another study in Saudi Arabia by Khubrani et al. (20) found that participants from different areas in the healthcare field (e.g., medicine, nursing, and dentistry) have an acceptable level of knowledge about IPC and HH. Students in the present demonstrated a significant improvement in their knowledge of IPC. Attitudes of students toward the implementation of IPC are very important and significant factors affecting compliance (21). In this study, although attitudes did not change because of attending the workshop, a significant improvement in students’ beliefs and commitment to the implementation of the IPC protocols was reported. However, these results were not reflected in the post-test I and post-test II levels of knowledge. Also, the results of those tests did not show significant adherence to IPC and HH protocols. The results of a systematic review of nurses’ compliance conducted by Nasiri et al. (22) showed that nurses in most showed positive attitudes toward the implementation of IPC guidelines and that these guidelines are important in protecting both the staff and the patients. This conforms to previous studies, which reported the same equal and moderate to positive attitudes exhibited by nursing students, such as a study conducted in Iran by Sarani et al. (23), and in Zambia by Chitimwango (24) and Mukwato et a. (25).

It was also found that students in this study had no significant differences between all academic levels based on their adherence and compliance with IPC and HH protocols. This finding is opposite to other studies, like Yusefi et al. (26), Timilshina et al. (27), which reported that nurses and nursing students differed in their intention to comply with IPC according to their clinical practice and the location of the hospital they were training in. However, a study by Nofal et al. (28) found no significant difference in the clinical setting, but rather the age and the length of clinical experience were predictors of knowledge and attitudes of nurses toward the implementation of IPC and HH protocols. Furthermore, this study found that students’ beliefs and commitment had improved significantly in both post-test I and II. This finding is congruent with Hassan’s (19) study, which reported that nursing students’ compliance in post-test significantly increased compared with pretest scores. Choi and Kim (18) found that nursing students’ awareness of IPC was the most significant factor influencing their intention to comply with IPCs. Therefore, it was suggested that adding IPC and HH material in the clinical courses and maintaining students’ awareness of the importance of these protocols to the safety of the patients is essential to improve compliance (18).

It was found in this study that one-third of nursing students failed to perform hand rubs with alcohol properly before contacting their patients, and the percentage of students, who did not comply or did not perform hand hygiene, was always high (31–55%). This finding is like Avsar et al. (10), who found that many nursing students had low skill levels in HH and only a limited number of them complied with hand hygiene using hand washing technique properly. Mahmoud et al. (29) reported that many nursing students did not view hand washing as essential while changing gloves between their patients. In contrast, Colet et al. (4) found that compliance rates were high for hand hygiene after changing the gloves (78%) and for hand washing between patients (75.8%). Rahiman et al. (21) concluded that nursing students thought that changing gloves between patients wasn’t necessary during the procedure even when it was very contaminated. Students in this study changed gloves frequently; however, they failed to change them as necessary (21).

Further, the number of students in the present study, who complied with using gloves correctly, decreased continuously over time compared with the first observation. On the contrary, Hassan (19) found that the compliance rate increased significantly in the post-test compared to the pretest rate. However, these results represent only a single set of post-tests that has been carried out directly after conducting an educational workshop, which explained the importance of adherence to IPC and HH protocols to students. Therefore, results from Hassan (19) could not reflect changes in the level of adherence and compliance over time. Limitations in practice could be due to the complexity of the procedure and decreased number of exposures as compared to other simple, more frequent procedures. The need to develop evidence-based scales that measure compliance in both students and nurses is present to ensure that learned skills are also maintained even after graduation (30).

Many reported studies addressed student compliance to IPC and HH in cross-sectional studies, which could not reflect students’ retention of knowledge and skills over time. Results from this study provide evidence on the retention rates and compliance levels of nursing students. Based on the results of this study, it is recommended that nurse educators include material that explains to students IPC and HH protocols in each clinical nursing course. Although students in this did not reflect improvement in all study scales, they could not always reflect this improvement in their practice. Therefore, it is recommended that clinical instructors keep students always reminded of the IPC and HH during their clinical and lab training. This could be achieved using a pretraining conference which is a brief meeting before each clinical day and testing IPC and HH at different times during student training. It is also recommended that future research addresses what and why students do not practice IPC and HH principles. In addition, it suggested that including these skills in the evaluation of those courses would be beneficial. As well, it is recommended that clinical managers raise awareness of clinical staff to adhere to IPC and HH and present a model to students.

Based on the findings of the present study, it is suggested that clinical coordinators and instructors include IPC and HH components in each clinical course, especially during the initial lab training before going to the clinical setting. In addition, clinical instructors are required to observe and follow specific checklists for the implementation of IPC and HH instructions during clinical training of students using clinical checklists tailored based on each clinical course objective. Finally, this study had limitations in its sampling technique, the size of the sample is limited. It is recommended to replicate the study on a greater number of nursing students. Nursing courses, and programs in the future.

## Conclusion

Nursing students learn infection prevention and control and hand hygiene in clinical courses to ensure the safety of the students and the patient. Therefore, addressing this topic in undergraduate nursing study is essential to promote safe practice after graduation. This study adopted a follow-up and observational design. The results reflected how students maintained the learned HPC protocols and what was not retained. Saudi students showed positive attitudes and acceptable levels of reported compliance with IPC and HH protocols despite the low levels of knowledge, especially in the pretest. The study affirmed that a positive attitude influences compliance with the IPC and HH. It can be concluded that better knowledge and positive attitudes are required, but this needs to be followed and emphasized as students train in the clinical areas. The results highlighted the need for continuous training and follow-up of nursing students on IPC and HH to enhance knowledge and increase retention and compliance with these protocols. Future studies could benefit from the results of this study by adding IPC and HH components in clinical courses and measuring how best such principles can be emphasized and reflected as an ongoing practice during their study and after graduation.

## Data availability statement

The raw data supporting the conclusions of this article will be made available by the authors, without undue reservation.

## Ethics statement

Ethical approval was obtained from the Ethics Committees of the participating institutes complying with the local ethical rules and regulations within the participating universities in the study based on the ethics approval granted by Alghad International Colleges for Applied Medical Sciences (AICAMS-346/RN/2018). The studies were conducted in accordance with the local legislation and institutional requirements. The participants provided their written informed consent to participate in this study.

## Author contributions

MA: Conceptualization, Data curation, Writing – original draft. LA: Investigation, Methodology, Writing – original draft. RA: Validation, Writing – review & editing. AA: Conceptualization, Writing – original draft.
